# A Novel Class I HDAC Inhibitor, AW01178, Inhibits Epithelial–Mesenchymal Transition and Metastasis of Breast Cancer

**DOI:** 10.3390/ijms25137234

**Published:** 2024-06-30

**Authors:** Xiangxiang Liu, Yawen Chen, Yang Li, Ying Shen, Shasha Dong, Jiang Tan

**Affiliations:** 1 The Key Laboratory of Molecular Epigenetics of Ministry of Education (MOE), Northeast Normal University, Changchun 130024, China; liuxx604@nenu.edu.cn (X.L.); 18302490298@163.com (Y.S.); dongss@nenu.edu.cn (S.D.); 2 The Institute of Genetics and Cytology, Northeast Normal University, Changchun 130024, China; chenyw017@nenu.edu.cn (Y.C.); liy234@nenu.edu.cn (Y.L.)

**Keywords:** HDACi, AW01178, breast cancer, metastasis

## Abstract

Epithelial–mesenchymal transition (EMT) refers to the transformation of polar epithelial cells into motile mesenchymal cells under specific physiological or pathological conditions, thus promoting the metastasis of cancer cells. Epithelial cadherin (E-cadherin) is a protein that plays an important role in the acquisition of tumor cell motility and serves as a key EMT epithelial marker. In the present study, AW01178, a small-molecule compound with potential therapeutic efficacy, was identified via in-cell Western high-throughput screening technology using E-cadherin as the target. The compound induced the upregulation of E-cadherin at both mRNA and protein levels and inhibited the EMT of breast cancer cells in vitro as well as metastasis in vivo. Mechanistically, AW01178 is a novel benzacetamide histone deacetylase inhibitor (HDACi) mainly targeting class I histone deacetylases. AW01178 promoted the transcription and expression of E-cadherin through enhancing the acetylation level of histone H3 in the E-cadherin promoter region, thereby inhibiting the metastasis of breast cancer cells. The collective findings support the potential utility of the novel HDACi compound identified in this study, AW01178, as a therapeutic drug for breast cancer and highlight its value for the future development of HDACi structures as anticancer drugs.

## 1. Introduction

Metastasis is the primary cause of cancer-related mortality. Depending on the type of tumor, metastasis often develops after a dormant period that can extend from several months to decades [1]. Metastasis is a complex cascade, starting with a phase of cancer cell dissemination from the primary tumor to regional and distal sites. Breast cancer is a heterogeneous disease that can be classified into four subtypes according to gene expression profiling (GEP): luminal, HER2+, basal-like, and normal breast-like [2,3]. Due to the heterogeneity of breast cancer, there are risks in diagnosis and therapy. The process of tumor metastasis is typically accompanied by the epithelial–mesenchymal transition (EMT) phenomenon [4]. EMT refers to the transformation of polar epithelial cells into motile mesenchymal cells under specific physiological and pathological conditions, which pathologically promotes fibrosis and cancer progression [5] and is generally considered the first step in metastasis [5,6].

Several studies have confirmed a significant association of epithelial cadherin (E-cadherin) with tumor metastasis. E-cadherin, a typical epithelial marker of EMT, regulates adhesion between cells [7,8]. Loss of the E-cadherin protein is necessary for cancer cells to undergo EMT and become invasive, while its re-expression during subsequent EMT progression plays an important role in survival and colonization for secondary metastasis [9,10]. Several transcription factors, such as Snail, Slug, ZEB1, ZEB2/SIP1, and Twist, have been shown to directly or indirectly repress E-cadherin promoter activity to induce EMT [11,12,13,14]. Recent studies have additionally reported that epigenetic modifications are involved in the regulation of E-cadherin, such as the recruitment of DNA methyltransferase (DNMT1) and histone deacetylase 1/2 (HDAC1 and HDAC2) by ZEB1 to the E-cadherin promoter to inhibit its expression [15,16].

Histone acetylation is catalyzed by two mutually antagonistic enzymes: histone acetyltransferases (HATs) and HDACs. Histone acetylation modification is a dynamic process whereby HATs are responsible for transferring acetyl groups of specific lysine residues of histones and HDACs for removing HAT-mediated acetylation. HDACs constitute a large family of highly conserved proteins. Human HDACs are divided into class I (HDAC1, 2, 3, and 8), class II (HDAC4, 5, 6, 7, 9, and 10), class III (also known as the Sirt family, Sirt1-7), and class IV (HDAC11) subfamilies based on their sequence homology to yeast orthologs. Abnormal expression of HDAC is significantly associated with a variety of disorders, including neurodegenerative and immune diseases, and the occurrence and development of cancer [17,18,19]. HDAC inhibitor (HDACi) compounds exert multiple biological effects in vivo, such as the induction of apoptosis and cell cycle arrest and the inhibition of tumor metastasis [8,20,21,22,23]. Suberoylanilide hydroxamic acid (SAHA) was the first HDACi to receive FDA approval for the treatment of advanced CTCL that failed to respond to multiple systemic treatment options [24]. Aminobenzamides act as selective inhibitors for class I HDACs. For example, MS-275 has been validated as a clinical treatment for multiple types of cancer [25,26]. However, several compounds under clinical development appear to have limitations, including serious adverse side effects, such as thrombocytopenia, fatigue, and diarrhea [27]. Although the results support an anticancer role of HDACi compounds in combination therapy with other agents, drug combinations face significant challenges, such as variations in drug solubility, which could lead to physical incompatibility, further promoting drug precipitation or inactivation [28]. Therefore, the screening and development of HDACi compounds is critical from a therapeutic perspective. 

In this study, using E-cadherin as the target, we attempted to screen compounds from small molecular libraries as well as newly synthesized small-molecule compounds with the aid of in-cell Western high-throughput screen technology. A novel benzacetamide compound, AW01178, was identified that exerted significant HDAC inhibitory activity and anticancer effects, both in in vivo and in vitro. Our collective results suggest that the suppression of metastasis of tumor cells through the inhibition of HDAC activity and the reversal of E-cadherin expression underlies the anticancer activity of a number of benzacetamide compounds, providing an important theoretical basis for the therapeutic effects of benzacetamide HDACi drugs.

## 2. Results

### 2.1. A Novel Small-Molecule Compound, AW01178, Upregulates E-Cadherin

**Figure 1:**
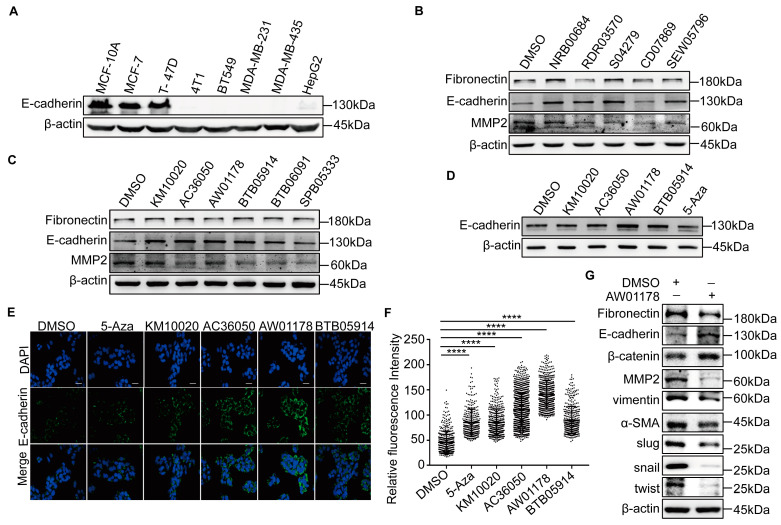
A novel small-molecule compound, AW01178, upregulates E-cadherin. (**A**) Western blot was used to analyze of the E-cadherin protein level in human breast cancer cells and liver cancer cells. (**B**,**C**) HepG2 cells were treated with 11 small-molecule compounds (10 µM), and western blot was used to analyze the epithelial marker E-cadherin and the mesenchymal markers MMP2 and fibronectin. (**D**) MDA-MB-231 cells were treated with 4 small-molecule compounds (10 µM), respectively, and western blot was used to analyze the epithelial marker E-cadherin. (**E**,**F**) MDA-MB-231 cells were treated with 4 small-molecule compounds (10 μM), and immunofluorescence was used to analyze the epithelial marker E-cadherin. Scale bar 100 µm (error bars indicate mean ± SD, *n* = 3 experimental replicates, **** *p* < 0.0001, Student’s *t*-test). (**G**) MDA-MB-231 cells were treated with AW01178 (10 μM), and western blot was performed to analyze the expression level of EMT markers.

E-cadherin was employed as the target to screen potential regulatory small-molecule compounds. We initially compared the expression levels of E-cadherin in epithelial mammary MCF10A, multiple breast cancer, and hepatoma cell lines. A Western blot analysis revealed lower E-cadherin expression in highly metastatic cell lines (Figure 1A). Two small-molecule compounds, trichostatin A (TSA) and 5-Aza, are reported to upregulate the expression of the E-cadherin protein. In keeping with this finding, the E-cadherin protein was upregulated in HepG2 cells treated with a range of concentrations of TSA and 5-Aza- 2′-deoxycytidine (Supplementary Figure S1A,B). To verify that in-cell Western technology can be applied to this experiment, we treated HepG2 cells with TAS and 5-Aza, respectively. The results showed that both TSA and 5-Aza could significantly upregulate the expression of E-cadherin (Supplementary Figure S1C,D).

To screen for small-molecule compounds that can upregulate the expression of E-cadherin, we applied for 320 kinds of organic small-molecule compounds in the drug-like compound library specially provided for high-throughput screening from the national major scientific research program of “development and reproduction research” of the Small-Molecule Compound Resource Center (https://www.screen.org.cn/ (accessed on 17 July 2016)) and 14 kinds of new organic small-molecule compounds synthesized by the Faculty of Chemistry, Northeast Normal University: these compounds are numbered 1–334. The ICW high-throughput screening technology showed that there are 11 small-molecule compounds that could upregulate the expression of E-cadherin among the 334 molecular compounds (Supplementary Figure S1E and Table S1). We also found that 4 compounds can upregulate E-cadherin at the protein level among the 11 compounds treated with HepG2 cells by the Western blot assay (Figure 1B,C). Western blot and immunofluorescence experiments were further applied to explore the effects of these four compounds in highly metastatic human MDA-MB-231 breast cancer cells. Among the test compounds, AW01178 induced significant upregulation of E-cadherin at both protein and immunofluorescence levels (Figure 1D–F). To further explore the impact of AW01178 on the breast cancer EMT process, epidermal and mesenchymal markers were detected by Western blot. The results demonstrated that the protein levels of E-cadherin and β-catenin were upregulated, and the expression levels of fibronectin, MMP2, vimentin, α-SMA, Slug, Snail, and Twist were decreased when treated with AW01178 (Figure 1G).

### 2.2. Small-Molecule Compound AW01178 Has the Ability to Inhibit Breast Cancer Migration and Invasion

**Figure 2:**
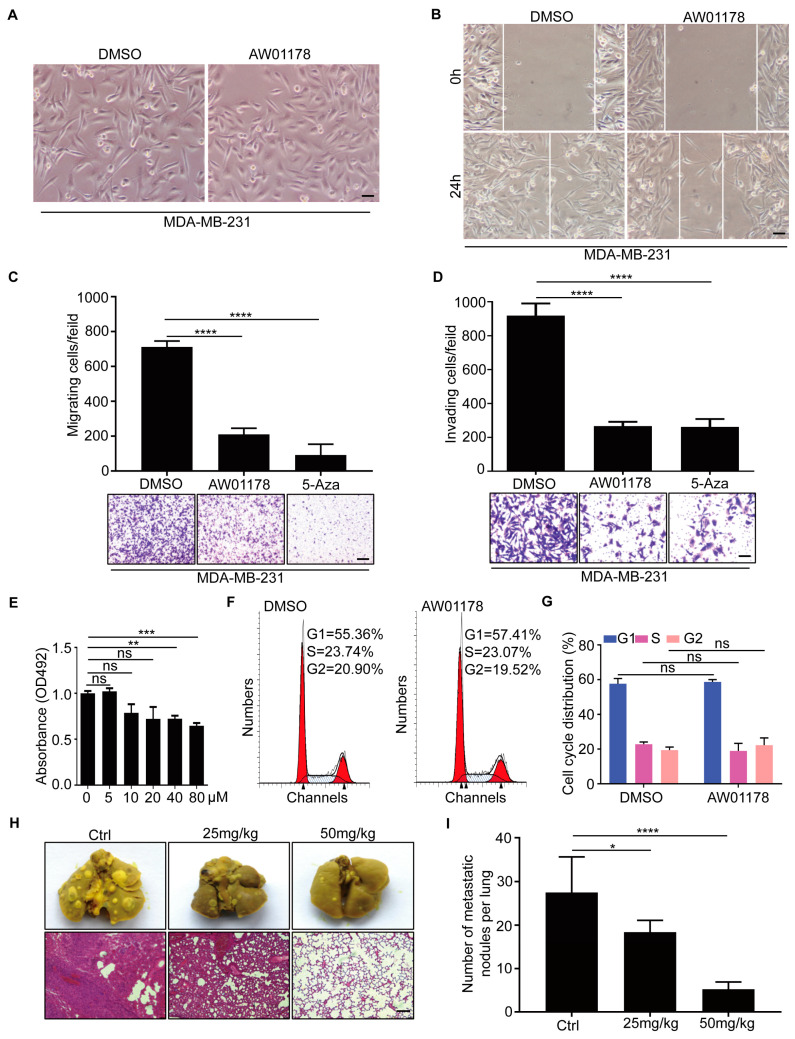
Small-molecule compound AW01178 has the ability to inhibit breast cancer migration and invasion. (**A**) Representative images of MDA-MB-231 cells treated with AW01178 (10 μM) or DMSO. Scale bars 100 μm. (**B**) Representative images of wound-healing assay of MDA-MB-231 cells treated with AW01178 (10 μM) or DMSO. Scale bars 100 μm. (**C**) Migration assays in MDA-MB-231 cells treated with AW01178 (10 μM) or 5-Aza (10 μM) or DMSO, respectively. Scale bar 100 µm (error bars indicate mean ± SD, *n* = 3 experimental replicates, **** *p* < 0.0001, Student’s *t*-test). (**D**) Invasion assays in MDA-MB-231 cells treated with AW01178 (10 μM) or 5-Aza (10 μM) or DMSO, respectively. Scale bar 100 µm (error bars indicate mean ± SD, *n* = 3 experimental replicates, **** *p* < 0.0001, Student’s *t*-test). (**E**) MCF-10A cells were treated with increasing doses of AW01178. The MTT assay was used to estimate the proliferation of cells (error bars indicate mean ± SD, *n* = 3 experimental replicates, ** *p* < 0.01, *** *p* < 0.001, *ns* = not significant, Student’s *t*-test). (**F**,**G**) MDA-MB-231 cells were treated with AW01178 (10 μM). Cell cycle assay was used to estimate the cell cycle (error bars indicate mean ± SD, *n* = 3 experimental replicates, *ns* = not significant, Student’s *t*-test). (**H**,**I**) Mice were killed and lung metastatic nodules were examined macroscopically or detected by H&E staining. Scale bar 100 µm (error bars indicate mean ± SD, *n* = 5 experimental replicates, * *p* < 0.05, **** *p* < 0.0001, Student’s *t*-test).

E-cadherin and matrix metalloproteinase-2 (MMP-2) are reported to be closely associated with tumor invasion [29]. Accordingly, we further evaluated the effects of the small-molecule compound AW01178 on metastasis of breast cancer. Following treatment of MDA-MB-231 cells with AW01178, cell morphology gradually changed from fibrous to cobblestone-like, representing an obvious reversal of the EMT process relative to the blank control group (Figure 2A). Subsequent data obtained from the wound-healing assay showed that the addition of AW01178 significantly reduced the migration speed of MDA-MB-231 cells compared with the control group (Figure 2B). In the Transwell migration and Matrigel invasion assays, AW01178 reduced the migration and invasion of aggressive MDA-MB-231 breast cancer cells, respectively (Figure 2C,D).

The objective of this study was to screen for small-molecule compounds that could specifically target and kill tumor cells without exerting side effects. The effects of different concentrations of AW01178 on the activity of MCF-10A, a human non-tumorigenic breast epithelial cell line, were evaluated via the MTT assay. Our results showed that high concentrations of AW01178 significantly inhibited cell activity (Figure 2E). At low concentrations, AW01178 significantly attenuated the invasion and migration ability of tumor cells, with no detectable effects on cell viability (Figure 2E) or the cell cycle (Figure 2F,G).

To explore the effect of AW01178 on breast cancer metastasis in vivo, 4T1 cells were injected into the tail veins of female nude mice. Two weeks after injection, mice were treated with AW01178 (25 or 50 mg/kg) or Ctrl, respectively, followed by dissection of lung tissues and examination via macroscopic and H&E staining after two weeks. Mice treated with AW01178 (50 mg/kg) cells formed fewer lung metastatic foci than those injected with Ctrl or 25 mg/kg AW01178 (Figure 2H,I). These findings strongly support a pivotal role of AW01178 in inhibiting breast cancer metastasis in vivo.

### 2.3. AW01178 Upregulates Acetylation of Histone H3 at E-Cadherin Promoter

**Figure 3:**
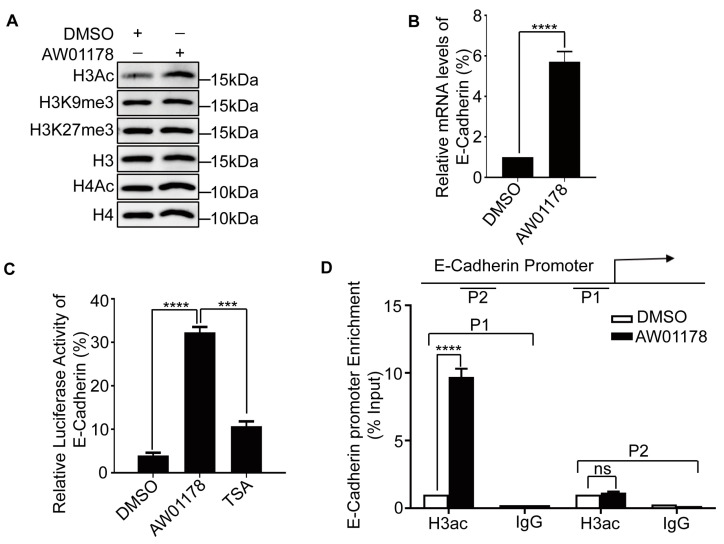
AW01178 upregulates acetylation of histone H3 at E-cadherin promoter. (**A**) MDA-MB-231 cells were treated with AW01178 (10 μM). Western blotting was used to measure histone expression. (**B**) MDA-MB-231 cells were treated with AW01178. Real-time PCR was used to measure E-cadherin expression. (**C**) MDA-MB-231 cells were treated with AW01178 (10 μM) and TSA (0.5 μM), respectively. The luciferase reporter assay was used to detect the expression of E-cadherin (error bars indicate mean ± SD, *n* = 3 experimental replicates, *** *p* < 0.001, **** *p* < 0.0001, *ns* = not significant, Student’s *t*-test). (**D**) qChIP results showing the increased H3Ac levels at the E-cadherin promoter after AW01178 (10 μM) treatment (error bars indicate mean ± SD, *n* = 3 experimental replicates, **** *p* < 0.0001, *ns* = not significant, Student’s *t*-test).

Accumulating evidence indicates that expression of E-cadherin is regulated by multiple epigenetic modifications, such as HDAC1/2, protein arginine methyltransferase 7 (PRMT7), histone demethylase LSD1, and coactivator-associated arginine methyltransferase 1 (CARM1) [5,30,31,32]. We further explored the mechanism by which AW01178 upregulates E-cadherin and inhibits the invasion and migration of tumor cells. Notably, in MDA-MB-231 cells treated with AW01178, a marked increase in the level of histone H3 acetylation was observed (Figure 3A).

AW01178 upregulated E-cadherin at translational levels (Figure 3B). Data from the luciferase reporter assay showed that AW01178 induced an increase in transcription from the E-cadherin promoter region (Figure 3C). In view of this finding, we examined the hypothesis that AW01178 affects the acetylation level of histone H3 in the E-cadherin promoter region, thereby increasing expression. To this end, the ChIP assay was conducted, which clearly demonstrated significant upregulation of the acetylation level of histone H3 at the E-cadherin promoter by AW01178 (Figure 3D).

### 2.4. AW01178 Is a Novel Class I HDAC Inhibitor

**Figure 4:**
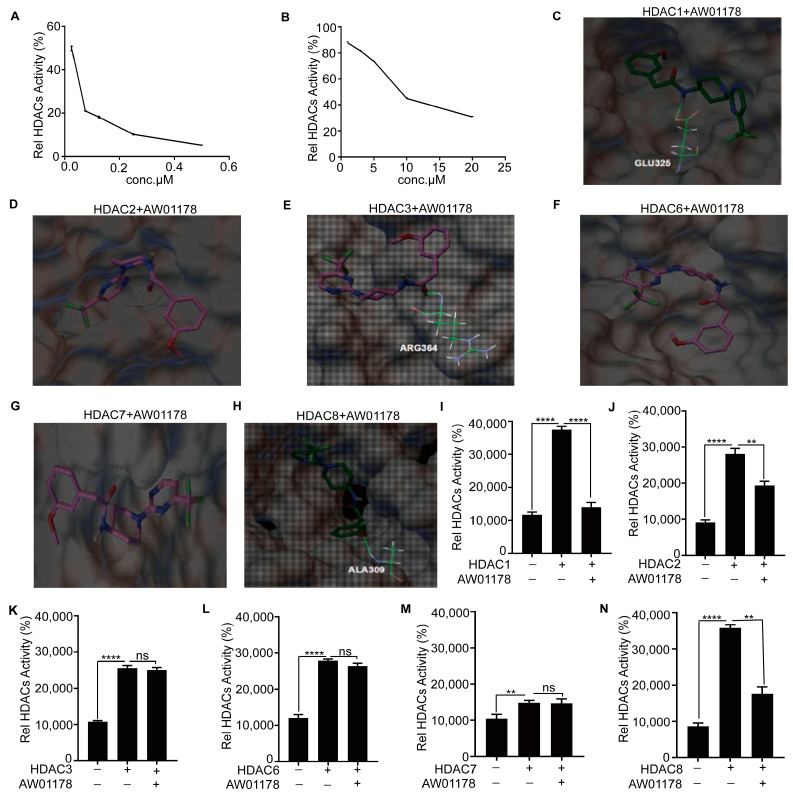
AW01178, a novel class I HDAC inhibitor. (**A**) An enzyme activity experiment was used to detect the relative enzyme activity of HDAC with increasing doses of TSA. (**B**) An enzyme activity experiment was used to detect the relative enzyme activity of HDAC with increasing doses of AW01178. (**C**–**H**) Autodock4.2.6 software was used to simulate a diagram of the compound AW01178 binding to various subtypes of HDAC (The background is different subtypes of HDAC, gray represents carbon atoms, red represents oxygen atoms, blue represents nitrogen atoms, yellow represents sulfur atoms; the thick stick model is compound AW01178, dark green or rose purple represents carbon atoms, blue represents nitrogen atoms, light green represents fluorine atoms, red represents oxygen atoms, and gray represents hydrogen atoms; the thin stick model represents an amino acid residue within the protein that forms hydrogen bonds with compound AW01178. Green represents carbon atoms, blue represents nitrogen atoms, white represents hydrogen atoms, and red represents oxygen atoms). (**I**–**N**) The effect of the small-molecule compound AW01178 (10 μM) on the activity of different subtypes of deacetylases (error bars indicate mean ± SD, *n* = 3 experimental replicates, ** *p* < 0.01, **** *p* < 0.0001, *ns* = not significant, Student’s *t*-test).

Several reports have shown that HDACi compounds, such as SAHA and TSA, induce the upregulation of E-cadherin, P21, and other genes, accompanied by abnormal histone acetylation or methylation levels at the promoter [33,34,35]. To ascertain whether AW01178 could function as an HDACi, we examined its deacetylase capacity. In vitro experiments performed using the deacetylase inhibitor TSA as a positive control showed suppression of HDAC activity by AW01178 in a dose-dependent manner (Figure 4A,B). Subsequent structural alignment analyses confirmed that AW01178 belongs to the benzacetamide class of HDACi. Simultaneously, Autodock4.2.6 software was applied to simulate the binding of AW01178 to various HDAC subtypes. The results showed that AW01178 was more likely to bind to class I HDACs (Figure 4C–H, Table S2). To further explore the inhibitory effect of AW01178 on HDAC activity, six deacetylases (HDAC1, HDAC2, HDAC3, HDAC6, HDAC7, and HDAC8) were purified in vitro, and a histone deacetylase activity detection assay was performed to detect the effects of AW01178 on the activities of purified HDACs. Our results showed inhibitory effects of AW01178 on the activities of HDAC1, HDAC2, and HDAC8 (Figure 4I–N). We further conducted drug interference experiments to eliminate the influence of drug interference on experimental results, which showed no interference of AW01178 with the detection signal (Supplementary Figure S2A). An examination of enzyme kinetics disclosed that the purified HDACs had enzymatic activities (Supplementary Figure S2B–G). Based on the collective results, we propose that AW01178 is a deacetylase inhibitor that specifically acts on members of the class I HDAC family, including HDAC1, HDAC2, and HDAC8.

## 3. Discussion

The E-cadherin target screening system used in this study is a valuable tool for screening HDACi compounds with anticancer activity. We successfully identified a novel benzacetamide small-molecule HDACi compound utilizing an ICW high-throughput screening technology-based E-cadherin target assay system. Four of the compounds screened induced upregulation of E-cadherin protein. Among these, AW01178, a novel benzacetamide compound, inhibited epithelial-to-mesenchymal transition. Furthermore, AW01178 exhibited significant intracellular HDAC inhibitory activity and suppressed the metastasis of breast cancer, both in vitro and in vivo. Remarkably, our findings showed that AW01178 presented similar inhibition effects in cell migration and invasion processes as 5-Azacytidine (Figure 2C,D), an epigenetic DNMT inhibitor, which has been widely applied in clinical situations [36]. Several studies have confirmed that combining HDACi with DNMTi can have beneficial effects, as the combination approach may increase the efficacy of each drug and stimulate the immune system to inhibit tumor progression [37]. Therefore, screening a novel and effective HDACi will be of significant benefit to anti-tumor treatment. 

HDACi compounds have a wide range of applications in the treatment of cancer, and multiple reports support the potential utility of these compounds in therapy for breast cancer. HDACi compounds have been shown to promote cancer cell cycle arrest and apoptosis [38,39,40], but can be associated with therapeutic resistance and toxic effects. However, AW01178, identified in this study, had no significant impact on the viability of normal epithelial cell MCF-10A other than at high concentrations. Our finding that AW01178 induces E-cadherin expression to a significant extent while exerting minimal toxic effects on normal epithelial cells (Figure 2E) supports its suitability for subsequent drug development.

The parent source of our novel small-molecule compound, AW01178, was 4-piperidic acid, which is widely used in the preparation of many drugs and bioactive substances, such as ropivacaine and the anti-tumor antibiotic sandramycin. Elucidation of the molecular mechanisms underlying the repair processes of E-cadherin proteins is critical for the development of therapeutic drugs. E-cadherin protein dysregulation is usually caused by abnormal histone methylation and acetylation. Methotrexate is reported to induce the downregulation of HDAC/EZH2, which can promote the methylation of histone H3 lysine 27, thereby increasing the expression of E-cadherin [41]. AW01178 did not significantly impact histone H3 lysine 27 expression but exerted a dramatic effect on H3ac that led to inhibition of metastasis (Figure 3A), supporting its potential as a novel anticancer drug. The compound may be further optimized to enhance therapeutic activity and modified for the development novel drugs. The combined effects of AW01178 with other anti-cancer drugs are yet to be explored.

Previous reports suggest that EZH2 plays a critical role in the control of cell invasion and/or metastasis by forming a co-repressor complex with HDAC1/HDAC2/Snail to inhibit E-cadherin [42]. Activated AR has been shown to cooperate with HDAC1, HDAC2, or HDAC3 to downregulate E-cadherin and promote the migration of non-metastatic breast cancer cells [43,44,45]. Based on these findings, it is inferred that HDAC1 and HDAC2 bind to the promoter region of the E-cadherin gene and that HDAC1, HDAC2, and HDAC3 play a crucial role in inhibition of E-cadherin expression. In this study, the small-molecule compound AW01178 was identified as a type I HDACi that could inhibit the deacetylase activity of HDAC1, HDAC2, and HDAC8, thereby affecting the acetylation level of histone H3 in the E-cadherin gene promoter region, in keeping with previous reports.

In conclusion, the discovery of AW01178, a novel HDACi, should greatly assist our understanding of the mechanisms of action of HDACi compounds. AW01178 significantly inhibits metastasis in breast cancer cells both in vivo and in vitro while exerting minimal toxicity and has promising prospects both as monotherapy and in combination with other anti-tumor drugs as a treatment agent for breast cancer.

## 4. Materials and Methods

### 4.1. Cell Culture

All cell lines were obtained from the American Type Culture Collection (Manassas, VA, USA) and characterized by DNA fingerprinting and isozyme detection. The MycoA-lert Mycoplasma Detection Kit (LT07-218; Lonza, Basel, Switzerland) was employed to ensure that all cell lines were negative for mycoplasma contamination every two months. A total of 320 types of organic small-molecule compounds were provided by the Small-Molecule Compound Resource Center, a major scientific research project of the National Development and Reproduction Research Program (Shanghai, China). AW01178 was purchased from Topscience (Shanghai, China, Cat#209302).

MCF10A cells were cultured as described in a previous report [5]. The 4T1 and BT549 cells were cultured in RPMI-1640 medium containing 10% FBS. MDA-MB-231 and MDA-MB-435 cells were cultured in L15 medium containing 10% FBS. MCF7, T-47D, and HepG2 cells were cultured in DMEM medium containing 10% FBS. Cell lines were cultured at 37 °C with 5% CO_2_, except MDA-MB-231 and MDA-MB-435 cells, which were cultured at 37 °C without CO_2_.

### 4.2. Reverse Transcription, PCR, and Real-Time PCR Analysis

Reverse transcription, PCR, and real-time PCR protocols were implemented as described in a previous study by our group [32]. The sequences of PCR primers are listed in the Supplementary Information.

### 4.3. Western Blotting

A Western blot assay was performed according to the standard protocol described in our earlier report [32]. The antibodies used are listed in the Supplementary Information.

### 4.4. Immunofluorescence

Immunofluorescence experiments were performed in keeping with previously reported guidelines [46].

### 4.5. Wound-Healing, Transwell Migration, and Invasion Assays

All experiments were performed as described previously [32]. For the wound-healing assay, cells were cultured in 6-well plates at a density of 1 × 10^6^ cells/well and treated with 10 μM AW01178 or DMSO. A 10 μL pipette tip was used to scratch cell layers, followed by the measurement of progression of cell migration. Transwell chambers with 8.0 μm polyethylene terephthalate membranes (24-well inserts, Corning BioCoat, Cat. No. 354166) were used for in vitro cell migration and invasion assays. For the migration assay, 2 × 10^4^ cells were cultured in the top chambers. For the invasion assay, 2 × 10^5^ cells were added to top chambers coated with Matrigel (BD Biosciences, Cat. No. 356234). Basic medium was added to the top chambers and complete medium added to the bottom wells in order to stimulate cell migration and invasion. Cells were stained with 0.1% crystal violet for visualization.

### 4.6. In-Cell Western Assays

Cells were cultured in 96-well plates at a density of 1 × 10^5^ cells/well and treated with 10 μM AW01178 or DMSO the next day. After 24 h of AW01178 or DMSO treatment, cells were fixed with 4% paraformaldehyde for 10 min and washed with PBS 3–4 times for 5 min. Subsequently, cells were treated with Triton X-100 (200 μL/5 min) and permeabilized more than four times. Cells were further incubated at 25 °C for 1.5 h with 5% BSA and washed with PBS 3–4 times for 5 min. Antibodies were separately added at the appropriate times, followed by fluorescence imaging with an Odessey instrument system and data processing.

### 4.7. Luciferase Reporter Assay

Experiments were performed as described previously [47]. The pGL4.15-E-cadherin (−420/+32) reporter construct was generated by Dr. Yao from our laboratory. 

### 4.8. Chromatin Immunoprecipitation

The chromatin immunoprecipitation assay was conducted in accordance with a previously published report by our group [48]. The primer sequences for the E-cadherin promoter were as follows: AGGGTCACCGCGTCTATG and CTTCCGCAAGCTCACAGG.

### 4.9. Cell Cycle

The cell cycle assay was implemented in keeping with previous reports [48]. Cells were treated with different concentrations of AW01178 or DMSO for 48 h, stained with propidium iodide (PI), and the cell cycle was examined via flow cytometry. 

### 4.10. MTT Assay

The MTT assay was performed as described previously [47].

### 4.11. Enzyme Activity Assay

The appropriate reagents were added to a 96-well plate in sequence according to the instructions of the histone deacetylase activity detection kit (Lot: BML-AK500-0001; Enzo Life Sciences; Farmingdale, New York, USA) and mixed well. Cells were incubated at room temperature for 30 min (3 replicate wells per reaction), followed by mixing with 50 μL 1 × Fluor de Lys ^®^ Developer (Lot: BML-AK500-0001; Enzo Life Sciences; Farmingdale, New York, USA) for termination of the reaction. Following incubation of the mixture for 15 min, a multifunctional ELISA reader (BioTek, Winooski, VT, USA) was utilized to detect fluorescence intensity, with parameters set to Ex. 350–380 nm, Em. 440–460 nm, and gain = 85.

### 4.12. HDAC Binding Analysis Assay

The three-dimensional structural files of various HDAC subtypes were downloaded from the Protein Data Bank (PDB) website. Three-dimensional structural files of the positive control TSA and compound AW01178 were constructed using Chem Draw v16.0.1.4 and Chem3D14.0.0.17 software.

### 4.13. In Vivo Mouse Lung Metastasis Assay

The details of the assay are provided in a previous study by our group [32]. First, 4T1 cells (1 × 10^6^ cells suspended in 150 μL PBS) were injected into the tail veins of 5-week-old female BALB/c nude mice (HFK Bioscience, Beijing, China). One week later, various doses of AW01178 or control were injected into the nude mice via intraperitoneal injection. After three weeks, the mice were sacrificed via euthanasia and their lungs were fixed in a paraformaldehyde solution for counting the number of metastatic lesions. The lungs were subsequently embedded in paraffin and H&E staining was performed. All animal experiments were approved by the Animal Care Committee of Northeast Normal University, Jilin, China.

### 4.14. Statistical Analysis

All results were obtained in triplicate from independent replicate experiments and presented as mean ± SD. Student’s *t*-test (two-tailed) was used to determine the significance of differences between groups, with *p* < 0.05 considered statistically significant. GraphPad Prism 7.04 Software (GraphPad Software, La Jolla, CA, USA) was employed for all statistical analyses.

## Data Availability

The data presented in this study are available in the Supplementary Material.

## Supplementary Materials

The supporting information can be downloaded at https://www.mdpi.com/article/10.3390/ijms25137234/s1.
