# Supplementary material for: A Novel Class I HDAC Inhibitor, AW01178, Inhibits Epithelial–Mesenchymal Transition and Metastasis of Breast Cancer
Source: Int J Mol Sci. 2024 Jun 30;25(13):7234. doi: 10.3390/ijms25137234 (PMC11241290; doi:10.3390/ijms25137234)
Supplement: Supplementary file 1 [file ijms-25-07234-s001.zip › Supplementary Information.pdf]

Supplementary Information for

A novel class I HDAC inhibitor, AW0118, inhibits epithelial-mesenchymal transition and metastasis of breast cancer

Supplementary Figures

Fig. S1. A novel small molecule compounds AW01178 that can upregulate the level of E-cadherin.

Fig. S2. AW01178 is a novel class I HDAC inhibitor.

Supplementary Figures

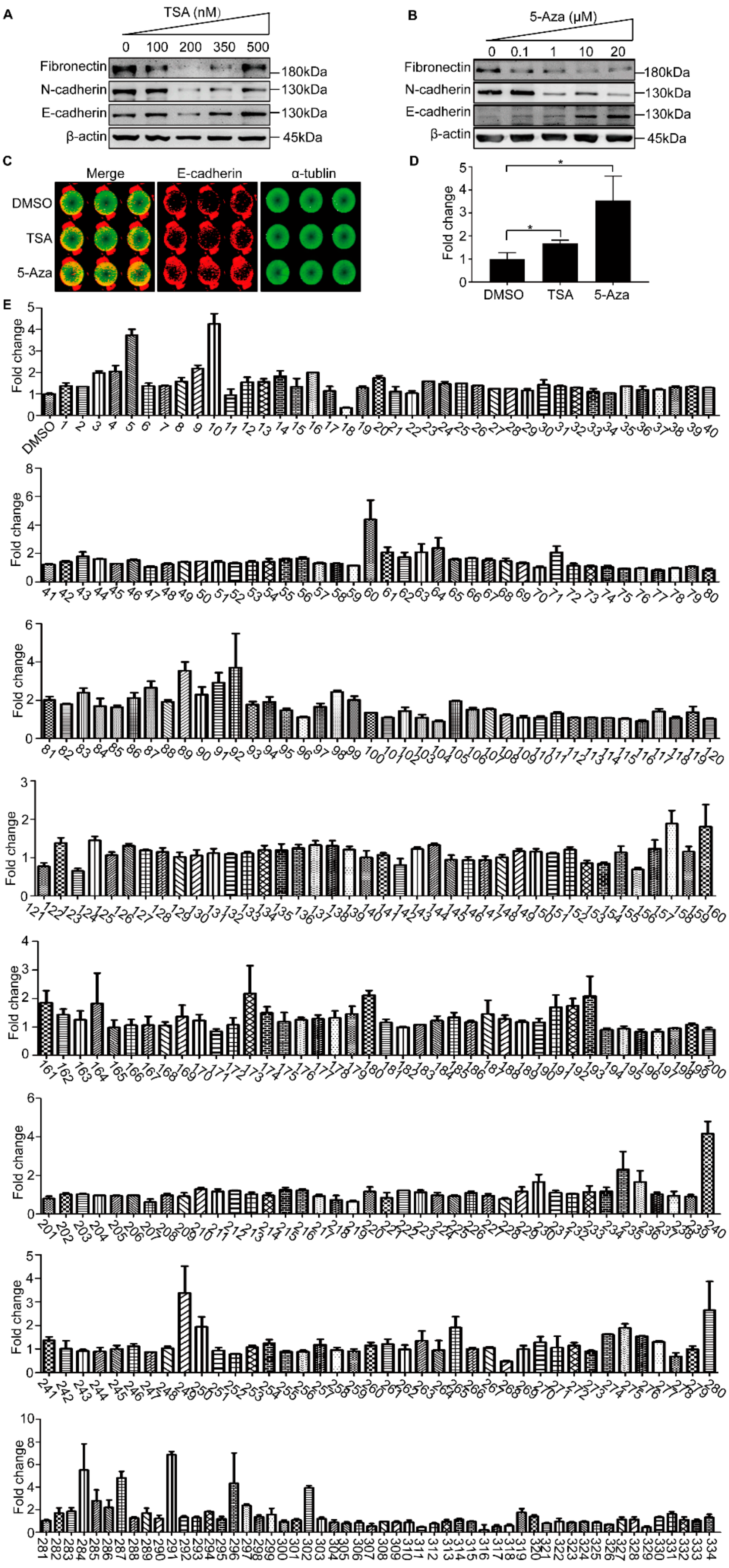

Figure S1. A novel small molecule compounds AW01178 that can upregulate the level of E-cadherin. (A) HepG2 cells were treated with increasing doses of TSA, respectively, and western blot was used to analyze the epithelial marker E-cadherin and the mesenchymal markers N-cadherin and fibronectin. (**B)** HepG2 cells were treated with increasing doses of 5-Aza, respectively, and western blot was used to analyze the epithelial marker E-cadherin and the mesenchymal markers N-cadherin and fibronectin. (**C-D)** HepG2 cells were treated with TSA (0.5 μM) or 5-Aza (10 μM), respectively. In-Cell Western assay was used to detect the expression of E-cadherin (error bars indicate mean ± SD, *n* = 3 experimental replicates, **P* < 0.05, Student's t-test). (**E)** HepG2 cells were treated with different small molecule compounds (10 μM), respectively. In-Cell Western assay was used to detect the expression of E-cadherin.

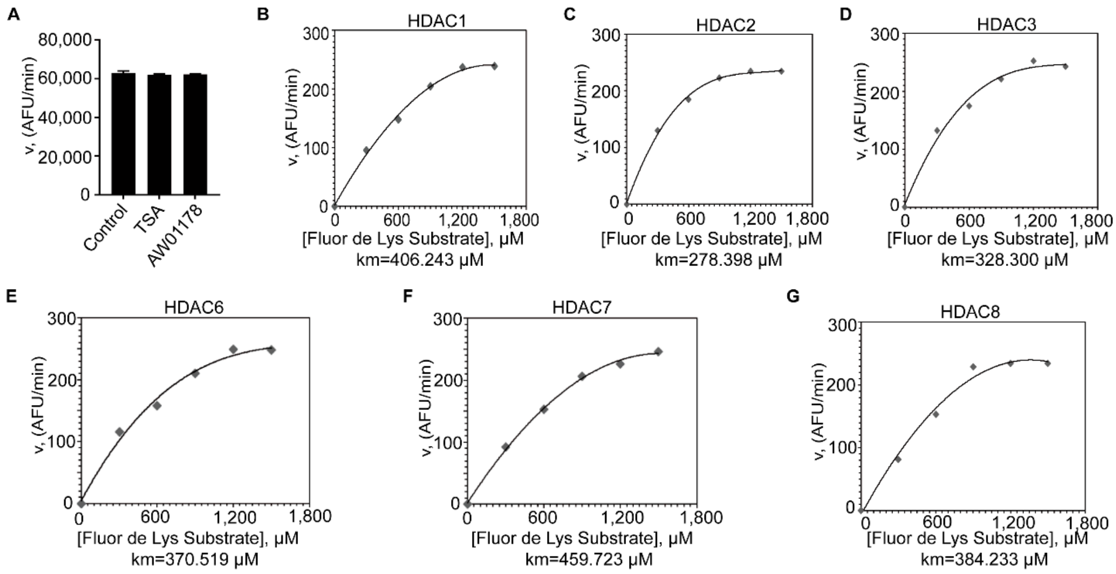

Figure S2. AW01178 is a novel class I HDAC inhibitor. (A) Drug interference experiments was used to eliminate the influence of AW01178 (10 μM) or TSA (0.5 μM) on the experimental results. **(B-G)** Enzyme activity experiment was used to detect relative enzyme activity of HDACs.

Table S1 The information of molecular compounds

Molecular structure	Sample number	Number	E-cadherin up-regulation
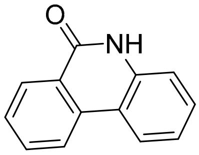	NRB00684	5	3.73
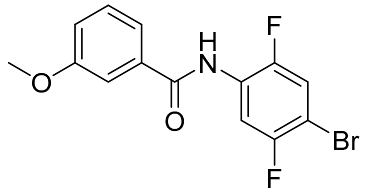	RDR03570	10	4.26
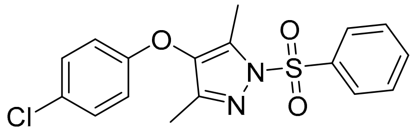	SPB05333	89	3.53
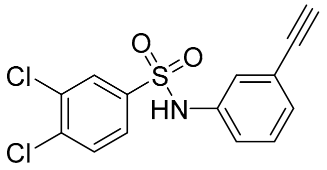	CD07869	92	3.70
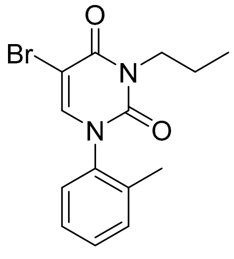	KM10020	240	4.17
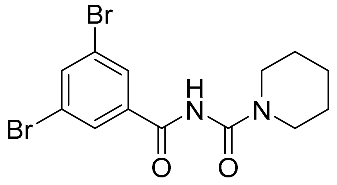	SEW05796	249	3.38
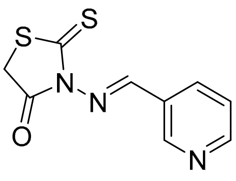	S04279	284	5.51
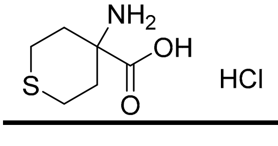	AC36050	287	4.81

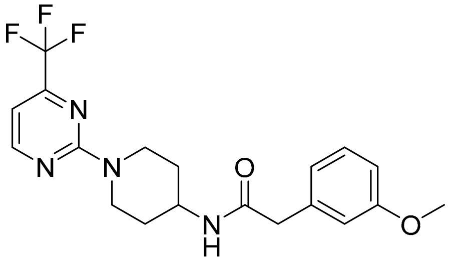	AW01178	291	6.86
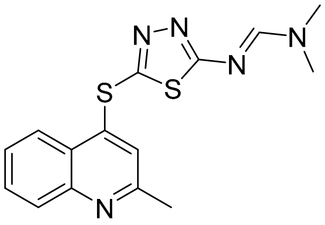	BTB06091	296	4.32
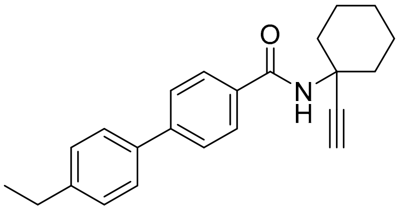	BTB05914	302	3.92

Table S2 Simulated data of compound AW 01178 binding to various subtypes of HDAC

Compound	AW01178		TSA	
Item	Binding free energy（kcal/mol）	Inhibition constant（μM）	Binding free energy（kcal/mol）	Inhibition constant (μM)
HDAC1	-5.24	143.21	-4.01	1160
HDAC2	-3.84	1530	-2.81	8680
HDAC3	-4.49	510.54	-3.25	4180
HDAC4	-4.61	419.94	-4.19	851.04
HDAC5	-3.85	1510	-3.28	3910
HDAC6	-4.1	986.6	-4.48	521.25
HDAC7	-4.82	290.69	-3.85	1520
HDAC8	-5.22	149.03	-3.72	1860

Supplementary Information for Materials and methods

1. Antibodies

Target Protein	Manufacturer	Item #	Host Species
β-actin	Sigma-Aldrich	A1978	Rabbit
acetyl-Histone H3	Millipore	06-599	Rabbit
acetyl-Histone H4	Millipore	06-866	Rabbit
H3	Abcam	Ab1791	Rabbit
H4	Abcam	Ab10158	Rabbit
N-cadherin	BD-Biosciences	610920	Mouse
E-cadherin	BD-Biosciences	610181	Rabbit
Fibronectin	BD-Biosciences	610077	Rabbit
MMP2	GeneTex GTX	104577	Rabbit
H3K9me3	Millipore	07-442	Rabbit
H3K27me3	Millipore	07-449	Rabbit

2. Reagents

REAGENT or RESOURCE	SOURCE	IDENTIFIER
L15	Sigma	Cat#L4386
DMEM	Sigma	Cat#S8112
1640	Sigma	Cat#R6504
DMEM/F12	Sigma	Cat#D8900
Fetal Bovine Serum	Selleckchem	Cat#S7132
Horse Serum	Gibco	Cat#16050-122
AW01178	Topscience	Cat#209302
TSA	Sigma-Aldrich	Cat#58880-19-6
5-Aza	Sigma-Aldrich	Cat#2353-33-5

3. qRT-PCR primer sequences

β-actin	sense	TCGTGCGTGACATTAAGGAG
	antisense	ATGCCAGGGTACATGGTGGT
E-cadherin	sense	AGGGTCACCGCGTCTATG
	antisense	CTTCCGCAAGCTCACAGG
