# Supplementary material for: A Novel Class I HDAC Inhibitor, AW01178, Inhibits Epithelial–Mesenchymal Transition and Metastasis of Breast Cancer
Source: Int J Mol Sci. 2024 Jun 30;25(13):7234. doi: 10.3390/ijms25137234 (PMC11241290; doi:10.3390/ijms25137234)
Supplement: Supplementary file 1 [file ijms-25-07234-s001.zip › Table S1.pdf]

Table S1 The information of molecular compounds

Molecular structure	Sample number	Number	E-cadherin up-regulation
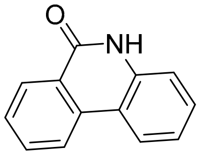	NRB00684	5	3.73
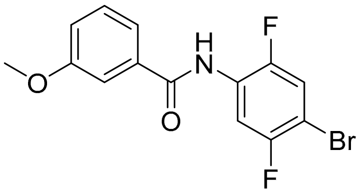	RDR03570	10	4.26
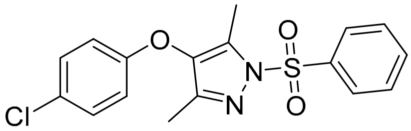	SPB05333	89	3.53
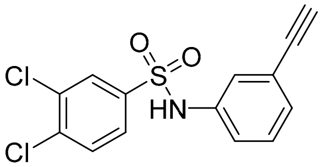	CD07869	92	3.70
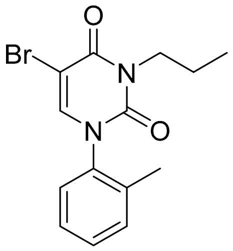	KM10020	240	4.17
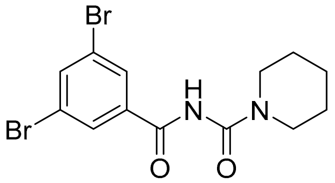	SEW05796	249	3.38
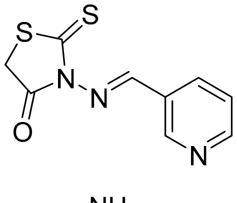	S04279	284	5.51
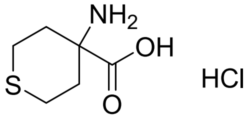	AC36050	287	4.81

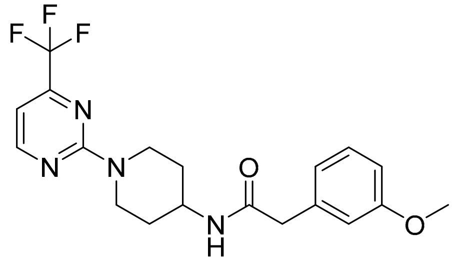	AW01178	291	6.86
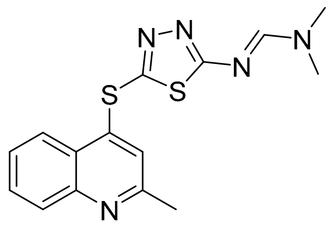	BTB06091	296	4.32
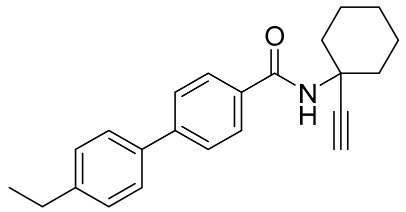	BTB05914	302	3.92
