# Supplementary material for: A Novel Class I HDAC Inhibitor, AW01178, Inhibits Epithelial–Mesenchymal Transition and Metastasis of Breast Cancer
Source: Int J Mol Sci. 2024 Jun 30;25(13):7234. doi: 10.3390/ijms25137234 (PMC11241290; doi:10.3390/ijms25137234)
Supplement: Supplementary file 1 [file ijms-25-07234-s001.zip › Table S2.pdf]

Table 2 Simulated data of compound AW 01178 binding to various subtypes of HDAC

Compound	AW01178		TSA	
Item	Binding free energy （kcal/mol）	Inhibition constant （μM）	Binding free energy （kcal/mol）	Inhibition constant (μM)
HDAC1	-5.24	143.21	-4.01	1160
HDAC2	-3.84	1530	-2.81	8680
HDAC3	-4.49	510.54	-3.25	4180
HDAC4	-4.61	419.94	-4.19	851.04
HDAC5	-3.85	1510	-3.28	3910
HDAC6	-4.1	986.6	-4.48	521.25
HDAC7	-4.82	290.69	-3.85	1520
HDAC8	-5.22	149.03	-3.72	1860
