# Supplementary figures and images for: A Novel Class I HDAC Inhibitor, AW01178, Inhibits Epithelial–Mesenchymal Transition and Metastasis of Breast Cancer
Source: Int J Mol Sci. 2024 Jun 30;25(13):7234. doi: 10.3390/ijms25137234 (PMC11241290; doi:10.3390/ijms25137234)

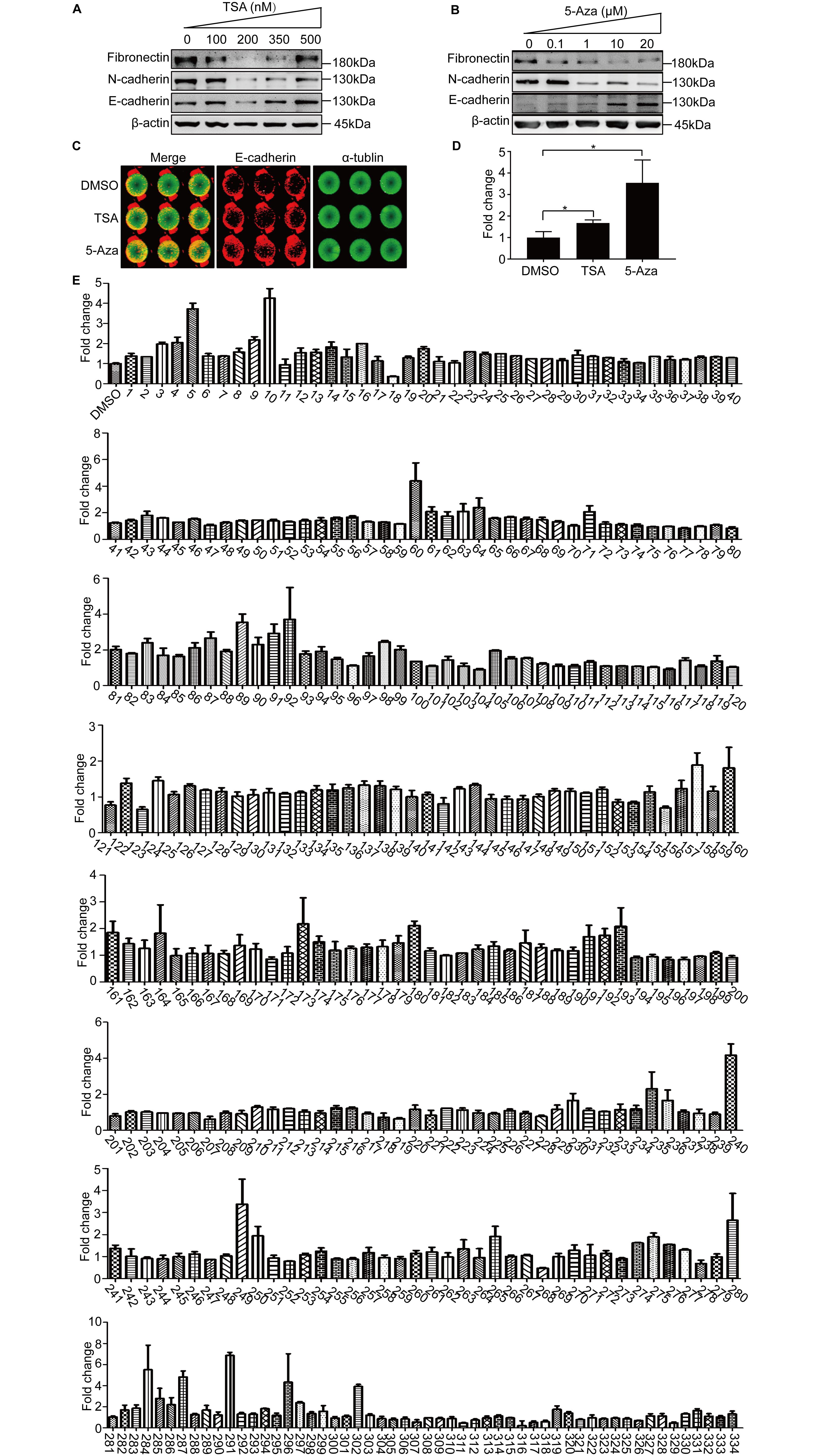

Supplement: Supplementary file 1 [file ijms-25-07234-s001.zip › Supplementary Figure S1.tif]

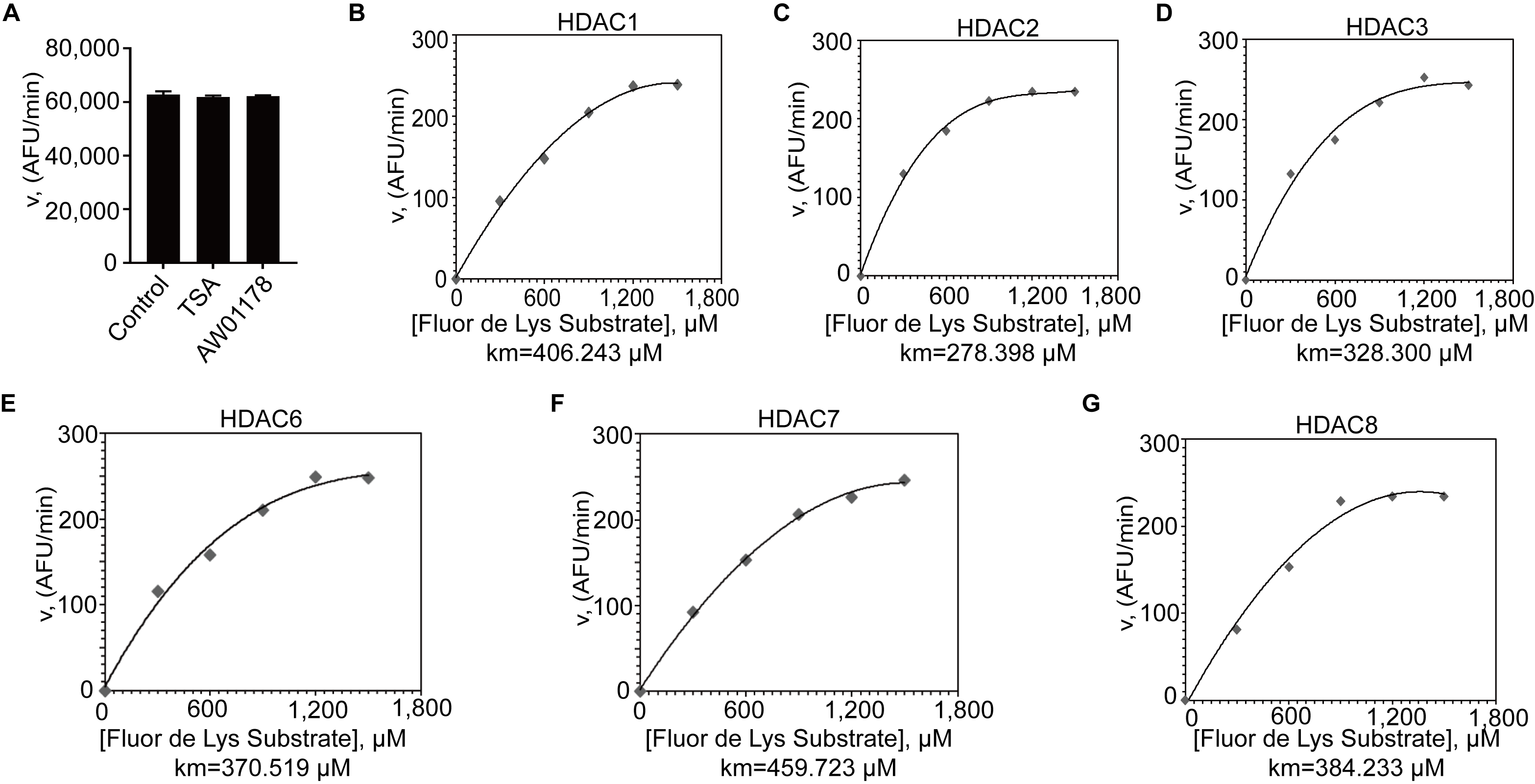

Supplement: Supplementary file 1 [file ijms-25-07234-s001.zip › Supplementary Figure S2.tif]
